# Oncostatin M promotes lipolysis in white adipocytes

**DOI:** 10.1080/21623945.2022.2075129

**Published:** 2022-05-16

**Authors:** Pim P. van Krieken, Julian Roos, Pamela Fischer-Posovszky, Stephan Wueest, Daniel Konrad

**Affiliations:** a Division of Pediatric Endocrinology and Diabetology, University Children’s Hospital, University of Zurich, Zurich, Switzerland; b Children’s Research Center, University Children’s Hospital, University of Zurich, Zurich, Switzerland; c Department of Pediatrics and Adolescent Medicine, Ulm University Medical Center, Ulm, Germany; d Zurich Center for Integrative Human Physiology, University of Zurich, Zurich, Switzerland

**Keywords:** Adipocyte, oncostatin M, lipolysis, glycoprotein 130, cytokine, insulin resistance

## Abstract

Oncostatin M (OSM) is a member of the glycoprotein 130 cytokine family that is involved in chronic inflammation and increased in adipose tissue under obesity and insulin resistance. OSM was shown to inhibit adipogenesis, suppress browning, and contribute to insulin resistance in cultured white adipocytes. In contrast, OSM may have a metabolically favourable role on adipocytes in mouse models of obesity and insulin resistance. However, a putative role of OSM in modulating lipolysis has not been investigated in detail to date. To address this, cultured white adipocytes of mouse or human origin were exposed to 10 or 100 ng/ml of OSM for various time periods. In murine 3T3-L1 cells, OSM stimulation directly activated hormone-sensitive lipase (HSL) and other players of the lipolytic machinery, and dose-dependently increased free fatty acid and glycerol release. In parallel, OSM attenuated insulin-mediated suppression of lipolysis and induced phosphorylation of serine-residues on the insulin receptor substrate-1 (IRS1) protein. Key experiments were verified in a second murine and a human adipocyte cell line. Inhibiton of extracellular signal-regulated kinase (ERK)-1/2 activation, abolished OSM-mediated HSL phosphorylation and lipolysis. In conclusion, OSM signalling directly promotes lipolysis in white adipocytes in an ERK1/2-dependent manner.

## Introduction

Oncostatin M (OSM) is an interleukin-6 (IL-6) type cytokine and member of the glycoprotein 130 (gp130) cytokine family. The OSM receptor complex is a heterodimer consisting of gp130 and the OSMRβ receptor subunit. Most actions ensuing from the binding of OSM to its receptor complex are mediated via the Janus kinase signal transducer and activator of transcription (JAK-STAT) signalling cascade, although depending on the cell type the extracellular signal-regulated kinase (ERK)-1/2 and the phosphatidylinositol‐3‐kinase (PI3K) cascades may also be involved [1].

OSM has various tissue-specific functions in physiological processes ranging from cancer cell proliferation and liver regeneration to cardiac remodelling and haematopoiesis [2]. OSM was also implicated in various aspects of adipose tissue (AT) biology and, in concert with other cytokines, contributes to AT homoeostasis [3,4]. Importantly, adipocytes do not produce OSM, but are highly responsive to the cytokine and abundantly express the OSM receptor complex [5,6]. Within the AT depot, resident immune cells such as macrophages and T-cells represent the main source of OSM [7].

OSM signalling in the adipocyte has been studied mostly in conditions characterized by chronic AT inflammation such as obesity and type 2 diabetes. The plasma and AT levels of OSM were higher in obese and/or insulin-resistant mouse models and humans compared with healthy controls [6,8,9]. Moreover, high-fat diet feeding upregulated *Osmr* gene expression in adipocytes of mice [7]. While these studies indicate that OSM signalling is increased in AT of obese and insulin-resistant individuals, it remains unclear whether OSM exerts metabolically protective effects or further perpetuates AT inflammation.

In support of the notion that OSM adds to adipocyte and metabolic dysfunction, OSM was shown to induce adipokines associated with insulin resistance and inflammation such as IL-6, plasminogen activator inhibitor-1 (PAI-1), and monocyte chemotactic protein‐1 (MCP-1) in cultured adipocytes [6,7,10]. OSM further contributes to the development of insulin resistance and obesity by inhibiting adipogenesis, lowering adiponectin expression, and suppressing the browning capacity of white adipocytes [4,9,11–14].

In contrast, global and adipocyte-specific OSMRβ knock-out studies demonstrated that the absence of OSM signalling in adipocytes aggravates body weight gain, adipose tissue inflammation and/or insulin sensitivity [5,7,15,16]. Treatment of obese mice with OSM was conversely accompanied by improvements in the above metabolic indices, and by reduction of circulating free fatty acid (FFA) and glycerol levels [15,17]. Importantly, an acute OSM challenge induced robust STAT3 and ERK1/2 phosphorylation in epididymal fat pads of wildtype mice, a response that was blunted in adipocyte-specific OSMRβ knock-out mice [16]. Interestingly, intraportal infusion of OSM repressed lipogenesis and induced lipolysis in the liver [15].

These *in vivo* studies suggest that OSM may be involved in AT lipolysis. Defined as the liberation of FFA and glycerol from stored triacylglycerols (TAG), lipolysis represents a chief function of white adipocytes that is activated during conditions of prolonged fasting or exercise. Mechanistically, cyclic AMP (cAMP)-mediated activation of protein-kinase A (PKA) initiates TAG hydrolysis by the consecutive actions of adipose triglyceride lipase (ATGL), hormone-sensitive lipase (HSL) and monoacylglycerol lipase (MGL), the former of which can be activated by perilipin (via comparative gene identification-58) and inhibited by G0/G1 switch gene 2 (G0S2) [18,19]. Though catecholamines and insulin are the main physiological promotor and suppressor of lipolysis, respectively, other factors such as cytokines can also modulate this process [20]. However, the lipolytic role of OSM was not investigated in detail to date and is the topic of the current paper. To avoid confounding paracrine interactions between adipocytes and immune cells or other components of the stromal vascular fraction, experiments were solely performed in cell culture models of white adipocytes.

## Results

### Prolonged exposure to OSM induces FFA release from adipocytes

**Figure 1.**
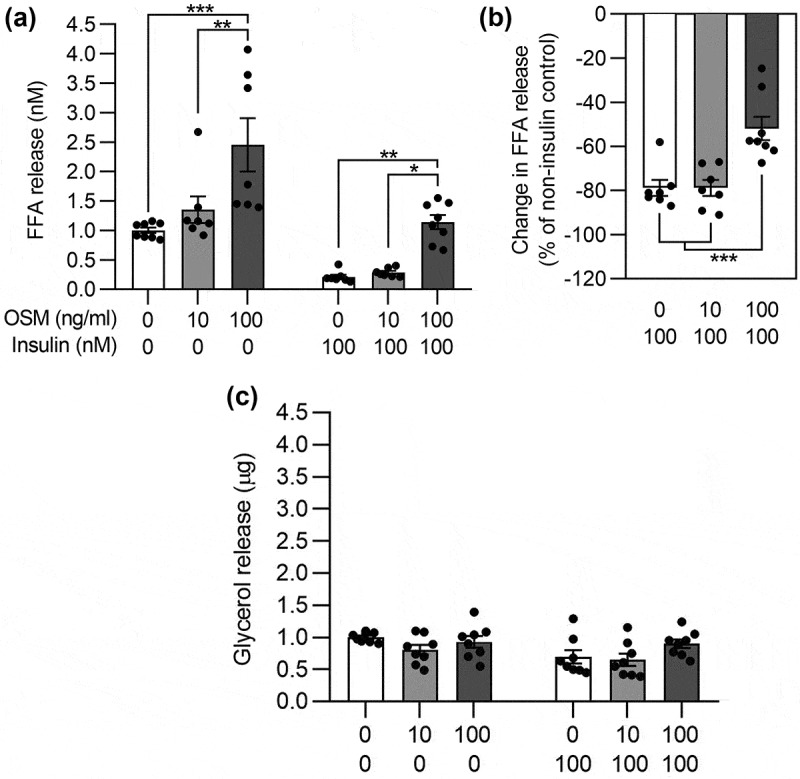
**Prolonged OSM stimulation induces free fatty acid release (FFA) from 3T3-L1 adipocytes**. (a) Relative FFA release in 3T3-L1 adipocytes stimulated with OSM and/or insulin. (b) Magnitude of the effect of insulin on FFA release expressed as a percentage of the corresponding OSM concentration without insulin. (c) Relative glycerol release in 3T3-L1 adipocytes stimulated with OSM and/or insulin. Cells were stimulated with OSM for 24 hours. FFA and glycerol were collected during the last 4 in the presence/absence of OSM and/or insulin (n = 7–8 from four independent experiments). Panel A, two-way ANOVA; panel B, one-way ANOVA, both with Tukey’s post-hoc test.

We previously found that prolonged (24 hours) stimulation of 3T3-L1 adipocytes with OSM lowered PPAR-γ levels at 100 ng/ml but not 10 ng/ml, while insulin-induced Akt phosphorylation was attenuated for both OSM concentrations [9]. To investigate the functional consequences of such changes, we assessed the effect of 24-hour OSM treatment on basal and insulin-inhibited lipolysis. OSM dose-dependently increased FFA levels by 1.4-fold and 2.5-fold for 10 and 100 ng/ml, respectively (Figure 1a). As expected, the addition of insulin decreased FFA secretion by approximately 80% in cells treated with vehicle. Whereas adipocytes treated with 10 ng/ml OSM responded similar to insulin as vehicle-treated cells, a dose of 100 ng/ml blunted the insulin effect to ~50% (Figure 1b), indicating that OSM induces insulin resistance dose-dependently. Conversely to the observed difference in the delivery of FFAs, no changes in glycerol release were detected after 24 hours of OSM treatment (Figure 1c).

*, *p* < 0.05; **, *p* < 0.01; ***, *p* < 0.001.

### OSM directly activates the lipolytic signalling machinery in white adipocytes

**Figure 2.**
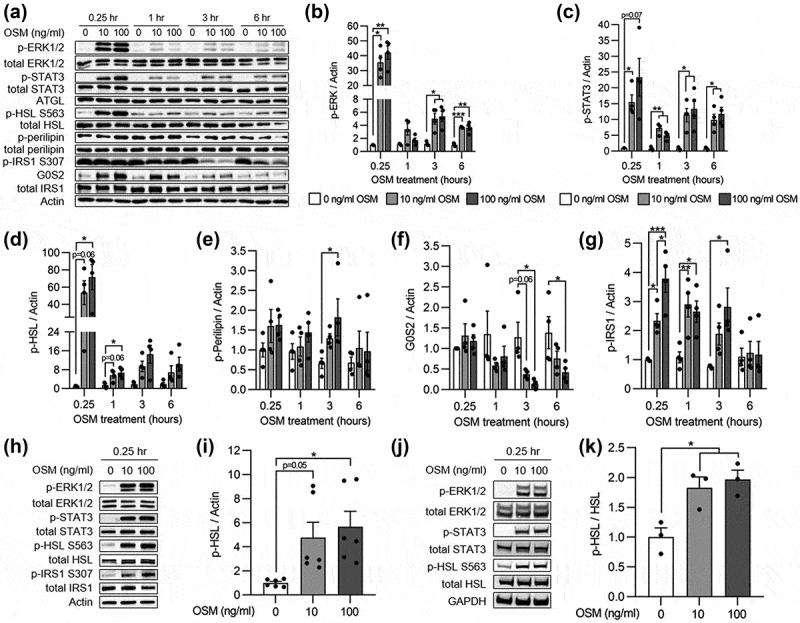
**Phosphorylation kinetics of lipolytic signalling mediators in adipocytes in response to OSM**. (a) Representative Western blots of 3T3-L1 adipocytes after treatment with OSM for 15 minutes or 1, 3 or 6 hours. (b-g) Relative expression of indicated phosphorlylated proteins and G0/G1 switch gene 2 (G0S2) at indicated time points after OSM treatment of 3T3-L1 adipocytes (n = 4 from three biological replicates; two-way ANOVA with Tukey’s post-hoc test). Representative Western blots of a subcutaneous murine cell line (h-i) and human SGBS adipocytes (j-k) stimulated for 15 minutes with OSM and the corresponding quantification of HSL phosphorylation relative to control (n = 6 and n = 3, respectively, both from three biological replicates; one-way ANOVA with Tukey’s post-hoc test). White, light grey and dark grey bars represent vehicle, 10 ng/ml OSM and 100 ng/ml OSM, respectively.

The observed effects of OSM on lipolysis could be direct or indirect, the latter for instance being mediated by OSM-induced expression of adipokines. To test the hypothesis that OSM affects lipolytic signalling directly, we looked at the phosphorylation kinetics of various signalling mediators in cells treated with OSM for 15 minutes, or 1, 3 and 6 hours (Figure 2a). Phosphorylation of STAT3 and ERK1/2, representing key downstream OSM signalling mediators in adipocytes, was significantly induced after OSM treatment (Figure 2b-c). Within 15 minutes of OSM stimulation, we further observed activation of HSL, followed by a rise in phosphorylated perilipin and a reduction in G0S2 after 3 hours (Figure 2d-f). These results suggest that OSM directly and acutely activates the lipolytic machinery in 3T3-L1 adipocytes.

*, *p* < 0.05; **, *p* < 0.01; ***, *p* < 0.001.

To investigate whether this effect of OSM is conserved between white adipocytes originating from different anatomical locations and species, additional experiments were performed in a subcutaneous murine cell line and in human Simpson Golabi Behmel syndrome (SGBS) adipocytes, also derived from the subcutaneous fat pad. OSM-mediated HSL phosphorylation at 15 minutes could be verified in both cell lines (Figure 2h-k), albeit to a lesser degree than what was observed for 3T3-L1 adipocytes.

In parallel, OSM increased phosphorylation of IRS-1 on serine 307 (Ser^307^) (Figure 2g-h). Since Ser^307^ phosphorylation of IRS-1 is linked to cytokine-mediated insulin resistance [21,22], it may explain the blunted effect of insulin on Akt phosphorylation [9] and on the reduction in FFA release (Figure 1b) in OSM-treated adipocytes.

### OSM promotes lipolysis in murine and human white adipocytes

**Figure 3.**
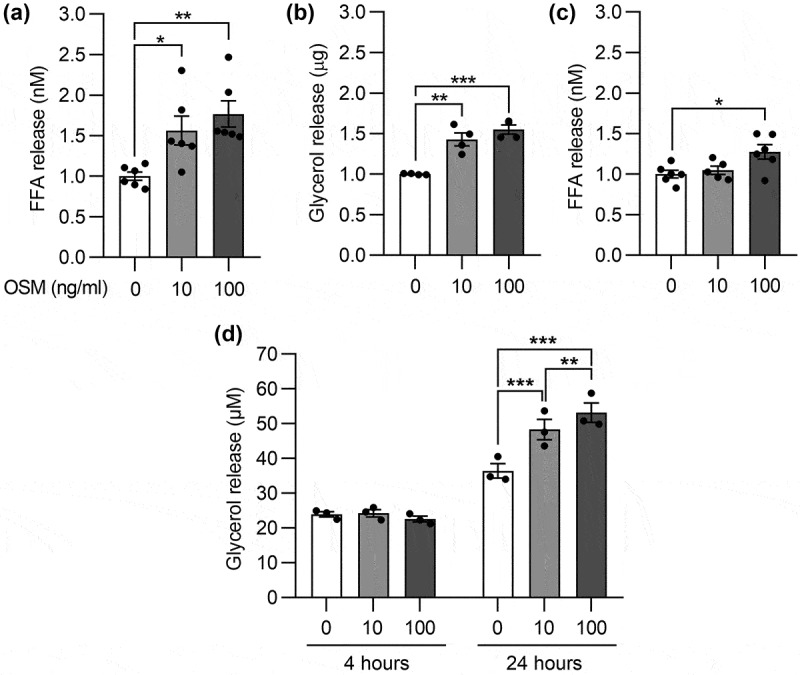
**OSM promotes lipolysis in murine and human white adipocytes**. (a-c) Relative free fatty acid (FFA) and/or glycerol release upon OSM stimulation of murine 3T3-L1 or subcutaneous adipocytes, respectively. FFA (n = 6) and glycerol (n = 4) were collected during the last 2 hours of a total of 3 hour treatment with the indicated concentrations of OSM and are presented as fold-change over control after correction for total protein content. (d) Glycerol release in OSM-stimulated human adipocytes collected during 4 or 24 hours (n = 3). Panel A-C, one-way ANOVA; panel D, two-way ANOVA, both with Tukey’s post-hoc test.

The above Western blot data spurred us to measure the pro-lipolytic capacity of OSM at a more acute interval. Concurring with the 24-hour stimulation protocol, FFA levels were significantly elevated after 3 hours of OSM treatment (Figure 3a). In contrast to the 24-hour time point, glycerol levels in the supernatant of OSM-treated 3T3-L1 adipocytes were also increased (Figure 3b). Such result could be reproduced in the murine subcutaneous adipocyte cell line when exposed to 100 ng/ml OSM (Figure 3c). In cultured human SGBS adipocytes, OSM did not elicit any changes in glycerol release during 4 hours of stimulation. However, a dose-dependent increase was measured in response to 24 hours of OSM exposure (Figure 3d). To verify that the elevated concentration of lipolytic products was not caused by possible cytotoxic effects of OSM, we assayed the supernatant for lactate dehydrogenase (LDH). The detected LDH levels were similar between control and OSM-treated 3T3-L1 cells (Supplementary Figure 1), excluding a potential cytotoxic effect of OSM as cause for the increased FFA/glycerol release. Jointly, these experiments indicate that OSM promotes lipolysis in white adipocytes, although the extent and kinetics may be different between species and cell lines.

*, *p* < 0.05; **, *p* < 0.01; ***, *p* < 0.001.

### Blocking ERK signalling prevents OSM-induced lipolysis

**Figure 4.**
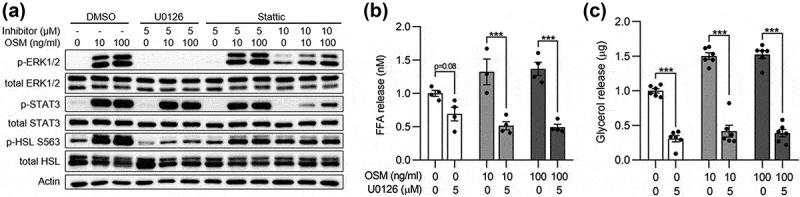
**ERK1/2 inhibition prevents OSM-mediated HSL phosphorylation and lipolysis**. (a) Western blots displaying ERK1/2, STAT3 and HSL phosphorylation in 3T3-L1 adipocytes stimulated for 15 minutes with OSM and pre-treated with DMSO (vehicle), U0126 or Stattic for 1 hour at the indicated concentrations. (b-c) Release of FFA (n = 3–4 from two biological replicates) and glycerol (n = 6 from three biological replicates) from 3T3-L1 adipocytes relative to controls. Cells were pre-treated with vehicle or 5 µM U0126 for one hour followed by three hours of OSM treatment. Lipolytic products were collected during the last 2 hours and are presented relative to controls after correction for total protein content. Panel B-C, two-way ANOVA with Šídák correction.

To test whether OSM-promoted lipolysis is mediated by ERK1/2 and/or STAT3, 3T3-L1 cells were treated with ERK1/2 or JAK-STAT3 pathway inhibitors 1 hour prior to OSM stimulation. The JAK2 inhibitor Tofacitinib dose-dependently prevented OSM-induced phosphorylation of ERK1/2 and moderately suppressed STAT3 phosphorylation at the highest concentration of 2 µM (Supplementary Figure 2A), thus acting as an inhibitor for both signalling pathways. The MEK inhibitor U0126 completely blocked OSM-induced phosphorylation of ERK1/2 at all concentrations tested without affecting STAT3 activation (Supplementary Figure 2B and Figure 4a). Stattic, an inhibitor of STAT3 activation, only partially prevented OSM-induced STAT3 phosphorylation at a dose of 10 µM. However, it also affected basal and OSM-induced ERK1/2 phosphorylation at this concentration (Figure 4a), disqualifying it as a specific inhibitor of the STAT3 pathway. Importantly, blunted OSM-induced ERK1/2 phosphorylation provoked by all three inhibitors was consistently associated with reduced phosphorylation of HSL, suggesting that OSM promotes lipolysis via ERK1/2 signalling. In support of such notion, pre-treatment with U0126 completely abolished the effect of OSM on FFA and glycerol release (Figure 4b-c). Of note, U0126 also blunted basal FFA and glycerol release, further supporting an important role for ERK1/2 in mediating adipocyte lipolysis. Nonetheless, we cannot exclude an additional role for STAT3 in OSM-induced HSL phosphorylation and subsequent lipolysis.

***, *p* < 0.001.

## Discussion

Multiple gp130 cytokines such as IL-6, leukaemia inhibitory factor and cardiotrophin-1 were previously shown to exert lipolytic activity [23–25]. Based on the structural similarities between OSM and its family members, Stephens and colleagues predicted that OSM would also have a pro-lipolytic capacity [20]. The present study provides evidence that OSM, at least *in vitro*, promotes lipolysis in white adipocytes.

Complementing data in murine and human white adipocytes indicates that the lipolysis-promoting properties of OSM are conserved between the two species. Yet, minor differences were found in the sensitivity to OSM, the extent of HSL phosphorylation and kinetics of lipolysis between the three cell lines. Various factors including the abundance of OSM receptors, differential negative feedback mechanisms, available lipolytic machinery, cross-talk between OSM and other cytokines, and the chosen experimental conditions may all contribute to an explanation for the discordances. Although the current study solely focused on *in vitro* models, a variable response to OSM may also be expected between distinct fat depots *in vivo*, since these likely harbour immune cell populations tailored to support their specific functions [26]. However, as exemplified below, a challenge with *in vivo* studies is to discern direct and indirect contributions of OSM to adipocyte function. By relying on cultured adipocytes that could be selectively exposed to OSM, we avoided confounding paracrine or endocrine signals such as those present in the context of a multicellular (inflammatory) AT environment. A limitation of our *in vitro* models is that they do not reproduce the immune cell-adipocyte interplay nor can these address how such an important relationship changes under conditions of low-grade AT inflammation.

We find that glycerol release in 3T3-L1 adipocytes was increased in response to 3 but not 24 hours of OSM exposure, while FFA delivery was elevated in both conditions compared to controls. We do not have a clear explanation for such finding; however, a previous publication mentioned (without showing the data) that 24-hour OSM stimulation did not alter glycerol release in a brown adipocyte cell line [12]. We speculate that OSM-induced lipolysis over time might have lowered substrate availability or activated a negative feedback due to FFA and glycerol accumulation in the incubation buffer, causing the lipolytic capacity during the collection period to be suppressed. Alternatively, prolonged OSM exposure in adipocytes might drive a differential reuptake of FFAs and glycerol, or a shift towards re-esterification of glycerol instead of release.

Our inhibitor experiments suggest that activated ERK1/2 mediates OSM-induced lipolysis, although we were unable to exclude the involvement of STAT3. Besides the lack of specificity of Stattic, the interpretation of our results is complicated by the fact that U0126 lowered both OSM-stimulated and basal lipolysis. However, such outcome is not surprising considering that HSL was previously identified as a substrate of activated ERK1/2 [27]. In fact, in addition to signalling via the conventional cAMP-PKA-dependent pathway, β_3_-adrenergic agents may also activate HSL via the ERK pathway [28]. Further supporting the involvement of ERK1/2, lipolysis induced by the cytokines TNF-α and IL-6 was also blocked by MEK/ERK inhibitors [29–31].

In conjunction with its pro-inflammatory actions, OSM was associated with indices of insulin resistance [6,8]. Nevertheless, the mechanistic evidence linking OSM to impaired insulin signalling in the adipocyte is sparse. In the current and our previous work, we provide *in vitro* evidence that exposure of adipocytes to OSM directly induces insulin resistance: OSM attenuated Akt phosphorylation induced by insulin [9], blunted the inhibitory action of insulin on FFA release, and induced phosphorylation of IRS-1 at Ser^307^ in 3T3-L1 adipocytes. The latter counteracts insulin-mediated phosphorylation of IRS-1 tyrosine residues required for further downstream signalling [21,22] and confirms a previous study examining OSM signalling in a fibrosarcoma cell line [32].

Our cell culture data conflicts with a series of mouse studies reporting that treatment with OSM improved insulin sensitivity, while the absence of OSM signalling in adipocytes gave rise to insulin resistance [5,15–17]. The metabolic phenotypes observed in the whole-body OSMRβ knockout mouse and following OSM treatment were in part attributed to a shift in the polarized state of AT macrophages. Whereas a lack of OSM signalling induced conversion from the anti-inflammatory M2 to the pro-inflammatory M1 type, the opposite occurred following a week of OSM therapy [5,17]. Furthermore, in adipocyte-specific OSMRβ knockout mice, higher OSM levels and a worsened adipokine profile were found compared to controls [7,16]. This suggests that paracrine OSM signalling within the AT supports immune cell homeostasis and its disruption in the OSMRβ knock-out mice may have contributed to a number of adverse secondary effects, including the development of insulin resistance.

Lipolytic products from white adipocytes can serve as a fuel source for thermogenesis in beige and brown adipocytes [33,34]. We and others recently investigated a role of OSM in AT browning and thermogenesis and found that it inhibited such processes [9,12], thereby helping to conserve energy. Paradoxically, the current work indicates that OSM promotes lipolysis. Malignant tumours could plausibly benefit from this dual role of OSM due to increased adipocyte-derived substrate availability [35]. While greater OSM signalling in adipocytes might serve cancer growth, it should be noted that a direct action of OSM on cancer cells likely impairs their proliferative capacity [2]. Since AT macrophages represent an important source of OSM, we hypothesize that their polarization and/or activation state impacts on OSM secretion. In this regard, M2 polarization upon cold exposure was found to amplify thermogenesis and lipolysis pathways via catecholamine secretion [36]. More research on the role of OSM in the interplay between AT macrophages and adipocytes is warranted to improve our understanding of this complex topic.

In conclusion, we demonstrate that OSM can directly promote lipolysis in cultured white adipocytes. Our data further indicate that OSM impairs insulin action, which can indirectly contribute to enhanced lipolytic activity. Together with our previous findings on its role in suppressing white AT browning [9], OSM emerges as a contributor to the development of insulin resistance and obesity.

## Methods

### Cell culture

Reagents were from ThermoFisher Scientific unless otherwise specified. Murine pre-adipocytes were seeded in 12-, 24- or 96-well tissue culture plates coated with 0.1% gelatin (Millipore, ES-006-B) and grown until confluent at 37°C in a humidified atmosphere with 5% CO2 in complete medium (CM) containing 4.5 g/L glucose DMEM (31,966–021), 10% foetal bovine serum (FBS; SH30109.03), 100 IU/ml penicillin and 100 μg/ml streptomycin. Differentiation of 3T3-L1 cells was initiated 2 days after reaching confluency (day 0) using CM supplemented with 500 μM 3-isobutyl-1-methylxanthine (IBMX), 1 μM dexamethasone, 1.7 μM human insulin (Actrapid, Novo Nordisk Pharma AG) and 1 μM rosiglitazone. On day 3, cells were switched to CM containing 0.5 μM insulin for 48 hours (day 5). To complete differentiation, cells were cultured in CM with 2% FBS until day 7 and from here onwards maintained on CM containing 1 g/L glucose DMEM (21,885–025) and 2% FBS for at least two more days. Subcutaneous pre-adipocytes were differentiated using a similar protocol until day 5 and from then onwards maintained on 10% FBS medium as previously described [9]. The Sympson-Golabi-Behmel syndrome (SGBS) pre-adipocyte cell strain was cultured and differentiated to adipocytes as described [37,38]. In brief, adipogenesis was initiated upon reaching near confluency (day 0) using FBS-free DMEM-F12 (10,565–018) supplemented with 10 μg/mL transferrin, 20 nM insulin, 100 nM cortisol, 200 pM T3, 25 nM dexamethasone, 250 μM IBMX and 2 μM rosiglitazone (all from Sigma-Aldrich). On day 4, medium was changed to FBS-free DMEM-F12 supplemented with 10 μg/mL transferrin, 20 nM insulin, 100 nM cortisol and 200 pM T3.

### Oncostatin M and inhibitors

Mature 3T3-L1, subcutaneous or SGBS adipocytes were treated with 10 or 100 ng/ml recombinant mouse or human OSM (495-MO and 295-OM, R&D systems), or vehicle (0.1% w/v bovine serum albumin (BSA) in phosphate-buffered saline (PBS)) for the indicated durations. Tofacitinib (S5001, Selleck Chemicals), U0126 (ab120241, Abcam) and Stattic (S7024, Selleck Chemicals) were dissolved in DMSO (vehicle) and administered to CM at the indicated concentrations 1 hour before OSM treatment.

### Lipolysis

Mature 3T3-L1 or subcutaneous adipocytes were cultured for a total of 3 or 24 hours in the presence or absence of 10 or 100 ng/ml recombinant mouse OSM. During the last 2 or 4 hours, CM was replaced by stimuli-containing Krebs Ringer phosphate-HEPES buffer (KRH) supplemented with 0.1% fatty acid-free BSA after a brief rinse in warm PBS. Human insulin was added to KRH at a concentration of 100 nM as indicated in the figure legend. Commercially available kits were used to determine glycerol (F6428 and G7793, Sigma-Aldrich) and FFA (91898 and 91797, WAKO Chemicals) concentrations in KRH. Values were corrected for buffer volume and total protein content.

SGBS adipocytes on day 14 were incubated overnight in FBS-free DMEM-F12. For OSM treatment, culture medium was replaced with KRH supplemented with 1% fatty acid-free BSA and supernatant was collected 4 and 24 hours after stimulation with vehicle control or 10 or 100 ng/ml recombinant human OSM. The Glycerol-Glo assay (Promega J3150) was used to determine free glycerol in the supernatant.

### Lactate dehydrogenase

Lactate accumulation was measured from CM with the CyQUANT LDH Cytotoxicity Assay Kit (C20300, Invitrogen) and expressed as a cytotoxicity score according to the manufacturer’s instructions.

### Protein isolation and Western blotting

For the mouse cell lines, proteins were extracted in RIPA buffer supplemented with phosphatase and proteinase inhibitors. Protein concentration was determined with the Pierce BCA Protein Assay Kit (23225, ThermoFisher Scientific). Details on Western blotting procedures were previously described [9]. In brief, equal quantities of protein were resolved using SDS-PAGE and transferred onto nitrocellulose membranes, blocked in 5% dry fat milk, incubated with primary antibodies at 4°C and subsequently with corresponding secondary antibodies at room temperature. Developed membranes were imaged with a ChemiDoc MP Imaging System (BioRad) and quantified with ImageLab software (BioRad, version 5.2.1). The following primary antibodies were used: pERK1/2 Thr202/Tyr204, #9101; ERK1/2, #9102; pSTAT3 Tyr705, #9145; STAT3, #9132; ATGL, #2138; pHSL Ser563, #4139; HSL, #4107; Perilipin-1, #9349; IRS1, #2390 (1:1.000; all from Cell Signalling); p-Perilipin Ser522, #4856 (1:1.000; Vala Sciences); pIRS1 Ser307, #07-247 (1:1.000; Millipore); Actin, MAB1501 (1:2.500; Sigma-Aldrich). G0S2 antibody (1:1.000) was kindly gifted by Dr. X. Yang. Goat-anti-rabbit, ab6721 and mouse-anti-rabbit, ab6789 (Abcam) secondary antibodies were used at a dilution of 1:5.000.

For SGBS adipocytes, protein isolation and Western blot were performed as described previously [39]. The following primary antibodies were used: pERK1/2 Thr202/Tyr204, #9106; pSTAT3 Tyr705, #9131; STAT3, #9132; pHSL Ser563, #4139; HSL, #4107 (all from Cell Signalling); ERK1/2, #M5670 (Sigma-Aldrich); GAPDH, #12004168 (BioRad). The following secondary antibodies were used: StarBright Blue 520 goat-anti-mouse, #12005867; StarBright Blue 700 goat-anti-mouse, #12004158; StarBright Blue 520 goat-anti-rabbit, #12005870; StarBright Blue 700 goat-anti-rabbit, #12004162 (1:5.000, all from BioRad).

### Statistics

GraphPad Prism (version 8.0) was used to prepare graphs and perform one-way or two-way ANOVA with a Tukey or Šídák post-hoc test as indicated in the figure legends. Data were considered significant when the p-value was less than 0.05 and are presented as mean ± standard error.

## Supplementary Material

Supplemental Material

## Data Availability

The data supporting the findings of this study are available within the article, its Supplementary Information file or from the corresponding author upon reasonable request.
