# Supplementary material for: Oncostatin M promotes lipolysis in white adipocytes
Source: Adipocyte. 2022 May 16;11(1):315–24. doi: 10.1080/21623945.2022.2075129 (PMC9116407; doi:10.1080/21623945.2022.2075129)
Supplement: Supplemental Material [file KADI_A_2075129_SM2851.zip › supplementary/Supplementary Figure Legends.docx]

**Supplementary Figure Legends**

**Supplementary Figure 1 OSM does not have a cytotoxic effect on 3T3-L1 adipocytes**

Cytotoxicity score calculated using the instructions in a commercial lactate dehydrogenase (LDH) assay. LDH was measured in fresh cell culture medium of 3T3-L1 cells stimulated with vehicle or OSM (n=4).

**Supplementary Figure 2 Impact of Tofacitinib and U0126 on OSM-mediated HSL phosphorylation**

Western blots stained against phosphorylated and total ERK1/2, STAT3 and HSL using lysates from 3T3-L1 adipocytes that were pre-treated for 1 hour with the JAK2 inhibitor Tofacitinib (**A**) and the MEK inhibitor U0126 (**B**) followed by 15 minutes of OSM treatment at the indicated concentrations.
