# Supplementary figures and images for: Oncostatin M promotes lipolysis in white adipocytes
Source: Adipocyte. 2022 May 16;11(1):315–24. doi: 10.1080/21623945.2022.2075129 (PMC9116407; doi:10.1080/21623945.2022.2075129)

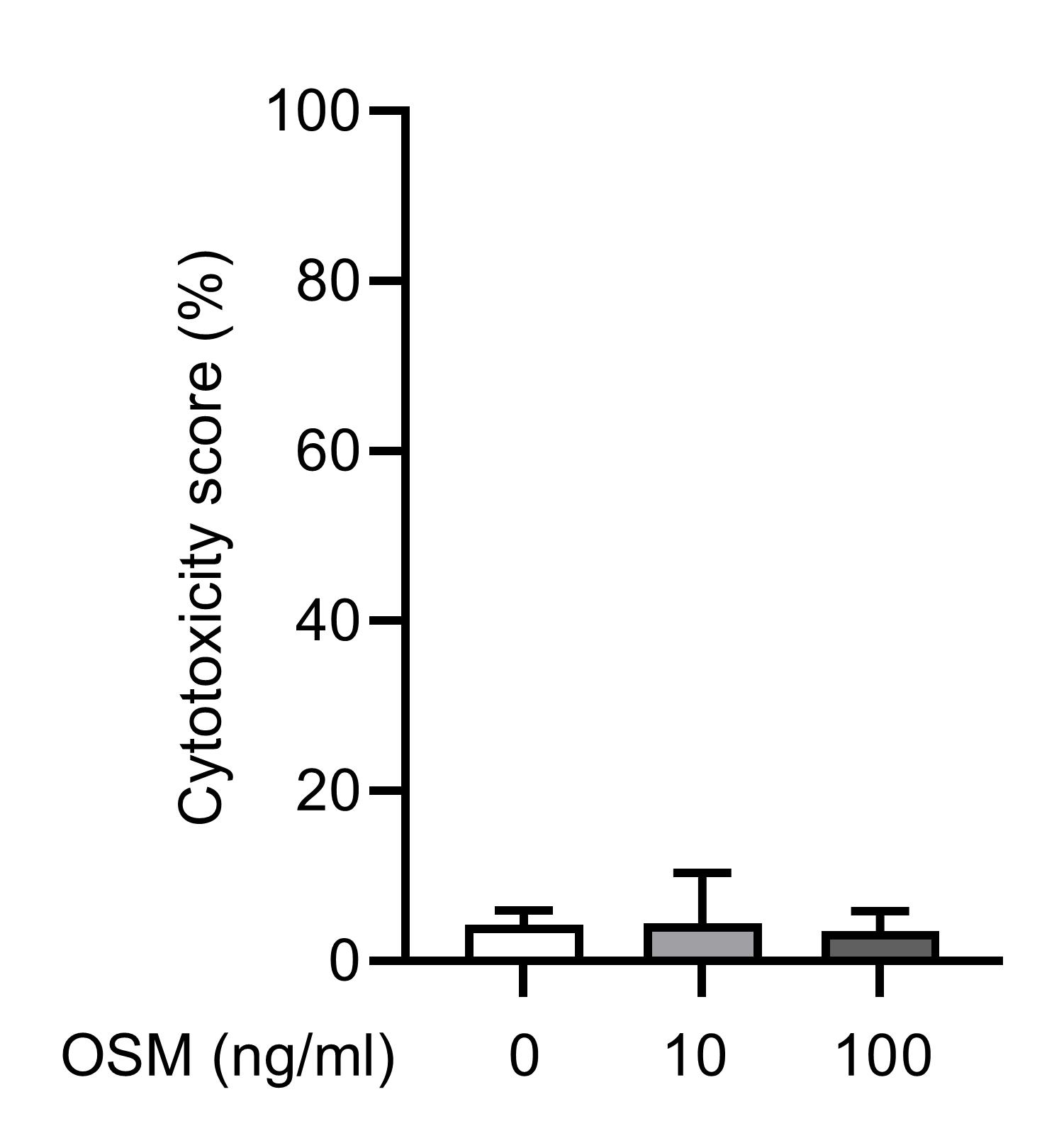

Supplement: Supplemental Material [file KADI_A_2075129_SM2851.zip › supplementary/Supplementary Figure 1.jpg]

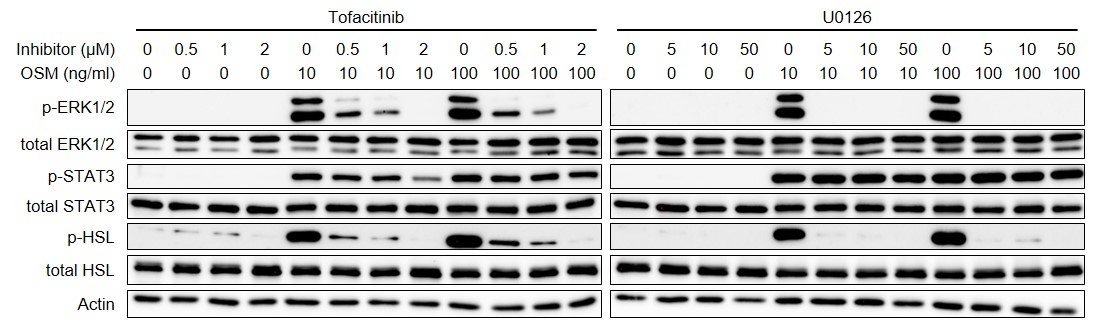

Supplement: Supplemental Material [file KADI_A_2075129_SM2851.zip › supplementary/Supplementary Figure 2.jpg]
